# Analysis of the Cerebrospinal Fluid Proteome in Alzheimer's Disease

**DOI:** 10.1371/journal.pone.0150672

**Published:** 2016-03-07

**Authors:** Payam Emami Khoonsari, Anna Häggmark, Maria Lönnberg, Maria Mikus, Lena Kilander, Lars Lannfelt, Jonas Bergquist, Martin Ingelsson, Peter Nilsson, Kim Kultima, Ganna Shevchenko

**Affiliations:** 1 Department of Medical Sciences, Cancer Pharmacology and Computational Medicine, Uppsala University, Uppsala, Sweden; 2 Affinity Proteomics, Science for Life Laboratory, School of Biotechnology, KTH Royal Institute of Technology, Stockholm, Sweden; 3 Analytical Chemistry, Department of Chemistry-BMC, Uppsala University, Uppsala, Sweden; 4 Department of Public Health/Geriatrics, Uppsala University, Uppsala, Sweden; University of Antwerp, BELGIUM

## Abstract

Alzheimer’s disease is a neurodegenerative disorder accounting for more than 50% of cases of dementia. Diagnosis of Alzheimer’s disease relies on cognitive tests and analysis of amyloid beta, protein tau, and hyperphosphorylated tau in cerebrospinal fluid. Although these markers provide relatively high sensitivity and specificity for early disease detection, they are not suitable for monitor of disease progression. In the present study, we used label-free shotgun mass spectrometry to analyse the cerebrospinal fluid proteome of Alzheimer’s disease patients and non-demented controls to identify potential biomarkers for Alzheimer’s disease. We processed the data using five programs (DecyderMS, Maxquant, OpenMS, PEAKS, and Sieve) and compared their results by means of reproducibility and peptide identification, including three different normalization methods. After depletion of high abundant proteins we found that Alzheimer’s disease patients had lower fraction of low-abundance proteins in cerebrospinal fluid compared to healthy controls (p<0.05). Consequently, global normalization was found to be less accurate compared to using spiked-in chicken ovalbumin for normalization. In addition, we determined that Sieve and OpenMS resulted in the highest reproducibility and PEAKS was the programs with the highest identification performance. Finally, we successfully verified significantly lower levels (p<0.05) of eight proteins (A2GL, APOM, C1QB, C1QC, C1S, FBLN3, PTPRZ, and SEZ6) in Alzheimer’s disease compared to controls using an antibody-based detection method. These proteins are involved in different biological roles spanning from cell adhesion and migration, to regulation of the synapse and the immune system.

## Introduction

Alzheimer’s disease (AD) is an age-dependent neurodegenerative disorder and the most common form of dementia in the elderly population, accounting for more than 50% of all dementia cases [1]. Epidemiological investigations have estimated that the numbers of AD patients will double every 20 years to more than 66 million worldwide in 2030 and 100 million by 2050 [2, 3].

Alzheimer’s disease is associated with multiple molecular characteristics including extracellular beta-amyloid (Aβ) plaque deposition and accumulation of intracellular neurofibrillary tangles composed mainly of hyperphosphorylated tau protein. These pathological findings are believed to mediate the extensive loss of neurons and synapses as well as the inflammatory processes [4]. The diagnosis of AD is based on clinical examinations that can be complemented by analysis of Aβ42, total tau (t-tau), and hyperphosphorylated tau (p-tau) level in cerebrospinal fluid (CSF) (reviewed in [5, 6]). Despite having relatively high sensitivity and specificity, these biomarkers have limited value for monitoring disease progression [6–8].

Cerebrospinal fluid is a proximal fluid in direct contact with the brain interstitial fluid that potentially reflects biochemical changes related to central nervous system (CNS), making it a promising source of biomarkers in neurological disorders such as AD [9]. CSF protein concentration can vary between 15 to 60 mg/dl and the protein level can be affected by age [10]. Over the last decade, there has been a growing interest in applying proteomics to identify disease-specific biomarkers to increase our understanding of underlying pathogenesis of AD. Most CSF biomarker discovery studies have been performed using a classic proteomics platform based on two-dimensional gel electrophoresis (2-DE) in combination with mass spectrometry (MS) or tandem mass spectrometry (MS/MS) [11–13]. Although 2-DE provides high resolution protein separation, it has limitations regarding detection of low abundant proteins [14]. As an alternative, gel-free shotgun MS in conjunction with quantitative proteomic technique, e.g. stable isotope labeling [15, 16] or label-free methods [17–20], have recently been used for identification and quantification of proteins involved in the pathogenesis of AD. Furthermore, combining gel-free shotgun MS approaches with protein depletion of high abundant proteins enables detection and quantification of low abundant proteins [21].

To analyze data sets generated by mass spectrometry-based methods, specialized software programs are commonly required. Commercial solutions are widely popular, mostly because of providing user-friendly environments whereas, open source programs offer more flexibility in terms of possibilities to modify existing algorithms. Careful selection of proper programs for data processing is crucial, since different programs have been shown to produce different and in some cases contradictory results (for reviews on this topic, see [22]and [23]). This inconsistency has been traced back to the application of different algorithms and improper choice of parameters (by the users) due to complex interface or lack of proper documentation (for more detailed description of different factors, see [24]). In addition to selection of software for initial data analysis, different methods of downstream processing and analysis such as normalization and statistical testing will influence the results. The basic assumption for many studies including CSF is that the protein concentrations in patients and healthy controls are similar; an assumption that is also reflected in global normalization methods used [15, 25, 26], however if this assumption is not correct it may have an negative impact on the final result.

The aim of this study was to detect novel protein markers that can be used to distinguish between AD and healthy elderly controls, to evaluate the consistency of software selection and impact of normalization methods used on result. We have employed a novel “Dot-it-Spot-it®” total protein assay to measure protein concentrations of small volumes of CSF from ten patients diagnosed with AD and ten non-demented subjects. The label free mass spectrometry data was processed using five different programs to evaluate quantification reproducibility and peptide identification performance. To evaluate the assumptions of normalization on the final results, we applied three different normalization algorithms and compared the results to that of using an affinity proteomics approach utilizing antibody suspension bead arrays of selected proteins. Finally, eight proteins were validated as different in expression between AD and controls.

## Materials and Methods

### Samples

This study was based on proteomic analysis of CSF from ten AD patients and ten non-demented controls. Samples were collected according to the recommended consensus protocol for CSF collection and biobanking [27] and obtained from the Uppsala Berzelii Technology Centre for Neurodiagnostics biobank at the Uppsala University Hospital. All patients underwent brain imaging, routine laboratory testing as well as neurological and cognitive examinations. The average Aβ42, tau, and p-tau in the AD patients were 420±117, 652±376, 132±112 (mean±SD ng/l), respectively. The control subjects had normal cognition according to their MMSE performance. The Regional Ethical Review Board in Uppsala, Sweden had approved the collection of CSF samples and the conducted research (Dnr 48–2005). The participants provided their written informed consent for research. The main clinical features of the patients are summarized in Table 1.

**Table 1:** Demographic information of patients and controls.

Patient	Case	Gender	Age, years	Duration of disease (years)	Age of onset (years)	Aß42 ng/l	tau ng/l	p-tau ng/l
1	AD	Male	78	2	76	350	54	420
2	AD	Male	81	2	79	310	1380	227
3	AD	Male	78	2	75	470	340	76
4	AD	Male	88	3	85	290	420	61
5	AD	Male	75	3	72	420	1100	128
6	AD	Male	76	2	74	411	732	93
7	AD	Male	84	3	81	700	555	76
8	AD	Male	75	3	72	459	697	80
9	AD	Male	77	16	60	347	540	83
10	AD	Male	82	1	80	444	703	78
11	Control	Male	89	-	-	**-**	-	-
12	Control	Male	89	-	-	**-**	-	-
13	Control	Male	88	-	-	**-**	-	-
14	Control	Male	87	-	-	**-**	-	-
15	Control	Male	88	-	-	**-**	-	-
16	Control	Male	89	-	-	**-**	-	-
17	Control	Male	88	-	-	**-**	-	-
18	Control	Male	88	-	-	**-**	-	-
19	Control	Male	88	-	-	**-**	-	-
20	Control	Male	88	-	-	**-**	-	-

### Sample collection and handling

CSF was collected at room temperature by aspiration lumbar puncture into polypropylene tubes. After collection, the samples were directly centrifuged at 1300 g for 10 minutes at +4°C to pellet any cell debris. After centrifugation, all CSF samples were visually inspected for blood contamination, frozen and stored at -80°C.

### Chemicals and reagents

Acetonitrile (ACN), methanol (MeOH), acetic acid (HAc), formic acid (FA), ammonium bicarbonate (NH_4_HCO_3_) were obtained from Merck (Darmstadt, Germany). Acetone, ethylenediaminetetraacetic acid tetrasodium salt dihydrate (EDTA), protease inhibitor cocktail, phosphate buffered saline (PBS), trifluoroacetic acid (TFA), triethyl ammonium bicarbonate (TEAB), sodium citratemonobasic, sodium dodecyl sulphate (SDS), and chicken ovalbumin were purchased from Sigma Aldrich (St. Louis, MO, USA). For tryptic digestion, iodoacetamide (IAA), urea and dithiothreitol (DTT) were obtained from Sigma Aldrich and trypsin/Lys-C mixture (mass spectrometry grade; Promega, Mannheim, Germany). Ultrapure water was prepared by Milli-Q water purification system (Millipore, Bedford, MA, USA).

### Multiaffinity immunodepletion

To enrich the low abundant proteins prior to nanoLC-MS/MS analysis, each CSF sample was depleted of the seven highly abundant proteins (albumin, IgG, alpha-1-antitrypsin, IgA, haptoglobin, transferrin, and fibrinogen) using a human Multiple Affinity Removal System (MARS Hu-7) 4.6 mm×50 mm LC column (Agilent Technologies, Palo Alto, CA, USA) connected to ÄKTA Explorer 100 HPLC system (Pharmacia Biotech, CA, USA) in the order according to S1 Table. Depletion was performed according to the instructions provided by manufacturer with the exception that Buffer A in the supplied kit was replaced with PBS buffer (10 mM NaH_2_PO_4_/Na_2_HPO_4_, 3 mM KCl and 137 mM NaCl, pH 7.4) and Buffer B was replaced with 50 mM citrate buffer, pH 2.3.

Briefly, an aliquot of 500 μL of each CSF sample was dried using an ISS110 Speedvac system ISS110 (Thermo Scientific, Waltham, MA, USA). The dried samples were reconstituted in 100 μL of Buffer A and injected at 0.25 mL/min into the column equilibrated at room temperature with Buffer A. Fractions of 1 mL were collected and the depleted CSF was obtained in fractions 3 and 4, which were pooled together. After freezing 10 μL for protein estimation the remaining volume of the 2 mL pool was concentrated down under vacuum prior to protein digestion and MS analysis. After 5 mL washing with Buffer A, the eluent was changed to the pH 2.3 buffer and the flow rate was increased to 1 mL/min. The captured CSF proteins were released from the column and collected in fractions 10–12 and pooled. The pools were adjusted to neutral pH with 1 M NaOH to a final volume of 4.2 mL, and used only for protein estimation. Afterwards, the column was washed with Buffer A before next sample was injected.

### Protein quantification

Total protein concentration of the two pools from each CSF sample was estimated by an ultrasensitive total protein assay, the Dot-it-Spot-it® protein assay (http://dot-it-spot-it.com; Maplestone AB, Knivsta, Sweden) according to the instructions provided. Aliquots from the pools were diluted 1/10-1/40 in 0.75% SDS, 10 mM TRIS buffer pH 7.5, 0.15 M NaCl and 0.02% NaN_3_. Human albumin (Sigma) was used for calibration in the range 0.32–10 ug/mL. The total protein content of the crude CSF samples was measured after 200-times dilution of the sample. The diluted samples were dispensed in 1 μL aliquots on the detection sheets in 3 replicates and rapidly dried. The sheets were then placed in a large well with 1 mL of detection solution and incubated for 4 min, followed by 4 min incubation in 1 mL of washing solution. The absorbent sink was removed from the sheets. The sheets were then dried and mounted on the scanning template, which was detected by an Epson Expression 1600 Pro scanner (Epson, Long Beach, California, USA). The blackness intensity was quantified in each dedicated spot on the image with Image J (http://rsbweb.nih.gov/ij/). Protein concentrations were estimated by comparing the sample results with the outcome of the human albumin calibration curve using Rodbard curve fitting in Image J. The percentage of protein in the depleted fraction was calculated as the %-ratio between proteins in the depleted fraction/proteins in (depleted + released fraction).

### Protein digestion

The entire amount of protein in the depleted CSF sample was digested with trypsin. Briefly, the proteins were re-dissolved in 50 μL of digestion buffer (6 M urea, 100 mM TEAB). A volume of 10 μL of chicken ovalbumin solution (0.05 μg/μL) was added to each CSF sample. A volume of 15 μL of 45 mM aqueous DTT was added to all samples and the mixtures were incubated at 37°C for 2 hours to reduce the disulfide bridges. The samples were cooled to room temperature and 15 μL of 100 mM aqueous IAA was added before incubating the mixtures for an additional 40 min at room temperature in darkness to carabamidomethylate the cysteines. Afterwards, a volume of 50 μL of 100 mM TEAB was added to all the samples. Finally, trypsin/Lys-C mixture dissolved in 100 mM TEAB was added to the samples, yielding a final trypsin/protein concentration of 5% (w/w). The tryptic digestion was performed at 37°C overnight. Prior to mass spectrometry analysis, the peptides were purified and desalted on Isolute C18 solid phase extraction (SPE) columns (1 mL, 50 mg capacity, Biotage, Uppsala, Sweden) using the following schedule: The column was first wetted in 3×500 μL of 100% ACN and equilibrated with 3×500 μL 1% HAc. The tryptic peptides were adsorbed to the media using five repeated cycles of loading. The column was washed using 3×1 mL of 1% HAc and finally the peptides were eluted in 300 μL 50% ACN, 1% HAc. After desalting, the eluate was vacuum centrifuged to dryness and re-dissolved in 60 μL 0.1% trifluoroacetic acid prior to nano-LC-MS/MS.

### NanoLC-MS/MS analysis

The nanoLC-MS/MS experiments were performed using a 7 T hybrid LTQ FT mass spectrometer (ThermoFisher Scientific, Bremen, Germany) fitted with a nano-electrospray ionization (ESI) ion source. On-line nanoLC separations were performed using an Agilent 1100 nanoflow system (Agilent Technologies, Waldbronn, Germany). Each sample was analyzed by RP-nanoLC-MS/MS in duplicates (technical replicates) in the order according to S2 Table. The peptide separations were performed on in-house packed 15-cm fused silica emitters (75-μm inner diameter, 375-μm outer diameter). The emitters were packed with a methanol slurry of reversed-phase, fully end-capped Reprosil-Pur C18-AQ 3 μm resin (Dr. Maisch GmbH, Ammerbuch-Entringen, Germany) using a PC77 pressure injection cell (Next Advance, Averill Park, NY, USA). The injection volumes were 5 μL and corresponded to 2 μg of tryptic peptides. The separations were performed at a flow of 200 nL/min with mobile phases A (water with 0.5% acetic acid) and B (89.5% acetonitrile, 10% water, and 0.5% acetic acid). A 100-min gradient from 2% B to 50% B followed by a washing step with 98% B for 5 min was used. Mass spectrometric analyses were performed using unattended data-dependent acquisition mode, in which the mass spectrometer automatically switches between acquiring a high resolution survey mass spectrum in the FTMS (resolving power 50000 FWHM) and consecutive low-resolution, collision-induced dissociation fragmentation of up to five of the most abundant ions in the ion trap.

### Antibody suspension bead arrays

A bead-based microarray platform was used as an orthogonal method for analysis of proteins selected based on the mass spectrometry results. All the 20 samples and a technical triplicate represented by a sample pool were processed and analyzed as previously described [28]. In brief, crude CSF was diluted 1:2 and the protein content was labeled with biotin. In parallel, Human Protein Atlas antibodies (http://www.proteinatlas.org) generated towards the selected proteins were immobilized onto color-coded magnetic beads (Luminex corp.), each antibody assigned to a specific bead ID, and later combined into an array in suspension. The labeled samples were then further diluted 1:8 and heat treated at 56°C for 30 min followed by cooling to RT for 15 min before overnight incubation with the array. For readout, unbound proteins were washed off using a liquid handler (Biotek EL406) and detection mediated through a streptavidin-conjugated fluorophore (Invitrogen). At least 50 beads per identity were measured in a FM3D instrument (Luminex corp.) and the median fluorescence intensity (MFI) per bead and sample used for further analysis.

### Data analysis

#### Statistical analysis on CSF protein amount

The percentage of protein in the depleted CSF fraction was calculated as the ratio between protein amount in depleted fraction divided by the sum of amounts in both fractions, multiplied by hundred. A two-sample t-test was performed on percentage and amount of protein in depleted fraction as well as on the sum of total protein amount. The basic assumption of normally distributed residuals was not violated.

#### Mass spectrometry identification and quantification

The following five mass spectrometry data processing programs were used to perform identification and quantification: Sieve v 2.1 (Thermo), DecyderMS v2.0 (GE healthcare), Maxquant [29], PEAKS (Bioinformatics Solutions Inc.) and OpenMS [30]. The raw data was imported into the programs and retention time was cropped to the range from 1500 to 5400 seconds. The quantification was performed using the following parameters (default settings were used for unmentioned parameters): Sieve: low charge: 1, high charge: 4, retention time alignment window: 2 min, maximum number of frames: 7000; DecyderMS: ion peaks were automatically detected using a typical peak width of 0.4 min, signal to background threshold of 3, and uniform background subtraction. The resulting intensity maps were aligned using DeCyder MS 2.0, allowing a time tolerance of 2 min and m/z tolerance of 0.01 Da; Maxquant: Type: Standard, Multiplicity: 1, Match time: 1 min, alignment time window: 2 min; PEAKS: Retention time shift tolerance: 2 min, Mass error tolerance: 10 ppm; For OpenMS, we used an automated label free pipeline introduced by [31] using the following parameters: FeatureFinderCentroided: Mz tolerance: 0.07 Da, min spectra length: 6, max missing peaks: 3, slope bound of mass trace: 1, low charge: 1, high charge: 4, isotope Mz tolerance: 0.05; IDMapper: RT tolerance: 40 seconds, Mz tolerance: 10 ppm; MapAlignerIdentification: Mz: 10 ppm, RT: 120 seconds; FeatureLinkerUnlabeledQT: 10 ppm, RT: 60 seconds. The following software search engines were used to perform the identification: Mascot for Sieve and DecyderMS; Andromeda [32] for Maxquant; combination of SPIDER [33], PEAKS [34], and PEAKS DB [35] for PEAKS; and a combination of Xtandem [36] and omssa [37] for openMS (combined using “ConsensusID” [38] tool in openMS) using the default settings. For protein identification the UniProt/Swiss-Pro human database (release 2014_03, containing 20272 entries) with ovalbumin chicken protein sequence added to data database and combined with a decoy database (the sequences were reversed) was used; for identifying peptides the following settings were used for all the search engines: Enzyme: Trypsin, missed cleavages: 2 precursor mass tolerance: 10 ppm, fragment mass tolerance: 0.7 Da, minimum charge: 2, maximum charge: 3, fixed modifications: Carbamidomethyl (C), variable modifications: Oxidation (M) and Deamidated (N and Q). False discovery rate (FDR) was calculated based on the target/decoy database and the peptides with FDR lower than 0.05 were chosen as true positive hits (considering the risk of having one false positive in 20 observation). Peptides with FDR lower than 0.05 and log_2_ transformed data was used in all the subsequent analysis.

#### Software comparison

The five data analysis tools were compared in terms of number of identified and mapped peptides (peptides that were assigned to quantified features in MS intensity map), number of identified proteins, reproducibility between technical replicates (samples were analyzed in duplicate), unbiased separation of AD and healthy control using principal component analysis (PCA), and peptide level correlation to levels of the targeted proteins using antibody-based profiling.

To compare the number of identified peptides and proteins between the programs, only the peptides mapped to quantified features, with FDR lower than 0.05, and found in more than 90 percent of the replicates were included.

Reproducibility was measured as the coefficient of determination (R^2^) and variance ratio between the technical replicates calculated based on the peptides (non-normalized data) found in all the biological and technical replicates (full coverage) and across all software tested. The variance ratio for each sample (with two technical replicates, *t1* and *t2)* was given by:

$$variance\;ratio=\frac{\frac{\sum {\left(X_{t1}-\bar{X_{t1}}\right)}^{2}}{N_{t1}-1}}{\frac{\sum {\left(X_{t2}-\bar{X_{t2}}\right)}^{2}}{N_{t2}-1}}$$

Where *X*_*t*1_ and *X*_*t*2_ are the vectors of peptide intensities, $\bar{X_{t1}}$ and $\bar{X_{t2}}$ are the mean of peptide intensities, and *N*_*t*1_ and *N*_*t*2_ are the number of peptides quantified in the first and the second technical replicate, respectively. A variance ratio close to one was regarded as a low ratio. To evaluate technical reproducibility and measure distance between AD and control samples, the peptide intensities were transformed using PCA. Mahalanobis distance between the technical replicates as well as between AD and control samples was calculated based on the first two components of the PCA result. Hypothesis testing was performed using Mahalanobis distance and p-values were derived using F distribution, showing cluster similarity between the technical replicates as well as between AD and control samples (A higher p-value indicates more cluster similarity) [39].

#### Disease related proteins

To find proteins present with altered levels in AD patients compared to healthy controls, the data was first analyzed using reference normalization [40]. The correlation between the technical replicates was estimated using “duplicateCorrelation” [41] and the “lmFit” function applied to fit multiple linear models using the “limma” [42] package in R [43]. Finally, the “ebayes” function [44] was used to compute moderated t-statistics for comparison of AD versus control (assuming normal distribution of intensities). Using a liberal approach, peptides with p-value lower than 0.05 and with at least three observations (in 10 ADs and 10 controls) were selected for calculating proteins p-value and fold change (multiple isoforms of a proteins were regarded as different proteins). The significantly altered peptides (p-value <0.05) were manually curated based on the quality of quantified features, for OpenMS a specific cutoff of 0.2 for the feature quality score was used as quality cutoff and for remaining software all quantified features were visually inspected and incorrectly quantified or linked features were removed. For estimation of protein expression, the protein p-value was calculated as median of peptide p-values (as well as fisher's combined probability test [45]) and the protein fold change was calculated as median of peptide fold changes.

#### Antibody suspension bead arrays

The Wilcoxon rank sum test was applied using raw data for group wise comparisons based on the antibody suspension bead array data and p-values lower than 0.05 were regarded as statistically significant. The log_2_ fold change was calculated from the ratio of medians in the AD group over controls and used for comparisons to the mass spectrometry data. For proteins with multiple antibodies, the one with the lowest p-value was selected for the comparison.

#### Correlation between mass spectrometry and antibody-based profiling

In order to calculate the correlation between antibody-based profiling and MS the average of technical replicates was used. Pearson correlation coefficient between MS and antibody-based profiling was calculated between all the peptides (allowing no missing values) of a protein in MS and all the antibodies used for the corresponding protein in the antibody-based profiling method. The correlation was calculated for the raw and the three types of normalized MS data:

Median normalization: Intensity of the *i*^th^ peptides in the *j*^th^ sample (*p*_*ij*_) was subtracted by the median intensities of the all the peptides in the corresponding sample:

$$Normalized\;\left(p_{ij}\right)=\;p_{ij}-s_{i_{\text{median}}}$$

Where $s_{i_{median}}$ is the median of j^th^ sample.

Reference normalization: The normalized value of the *i*^th^ peptide in the *j*^th^ sample (*p*_*ij*_) was calculated as:

$$Normalized\;\left(p_{ij}\right)=\;p_{ij}-{\left(s_{j}-s_{ref}\right)}_{median}$$

Where (*s*_*j*_ − *s*_*ref*_)_*median*_ is the median of differences between sample *s*_*j*_ and a reference sample (*s*_*ref*_) which was selected as the sample with the lowest number of missing features.

Spiked-in normalization [40]: peptides of chicken ovalbumin protein (which was spiked in the samples as an internal standard) were used to compute normalization factor for each sample separately for each of the five programs. The chicken ovalbumin peptides were first mean-centered across the samples:

$$Normalized\;\left(cp_{ij}\right)=\;cp_{ij}-{cp}_{i_{mean}}$$

Where *cp*_*ij*_ is intensity of i^th^ chicken ovalbumin peptide in the j^th^ sample and ${cp}_{i_{mean}}$ is mean of the i^th^ peptide across all the samples. The peptide intensities in each sample were then subtracted by mean of the chicken ovalbumin intensities in the corresponding sample:

$$Normalized\;\left(p_{ij}\right)=p_{ij}-{cp}_{j_{mean}}$$

Where *p*_*ij*_ is the intensity of i^th^ peptide in the j^th^ sample and ${cp}_{j_{mean}}$ is mean of chicken ovalbumin peptides in the j^th^ sample.

For each antibody, the highest correlating peptide in mass spectrometry was selected to examine the effect of normalization on the correlations.

## Result

### CSF protein amount

The total amount of protein in the two pools obtained after the MARS Hu-7 column affinity purification of 0.5 mL CSF was 187±61 μg (mean±SD) for nine non-demented controls and 178±81 μg for ten AD patients, showing no significant difference. The result for one of the control samples was omitted, as the value of 587 μg was an outlier compared to the range 90–330 μg for the other 19 samples. Estimation of protein amount in crude CSF verified that this sample was aberrantly high compared to the other samples (we suspected that this sample was contaminated with blood and therefore, it was omitted only for statistical analysis performed on the protein amount but it was included in MS and antibody-based analysis).

**Fig 1:**
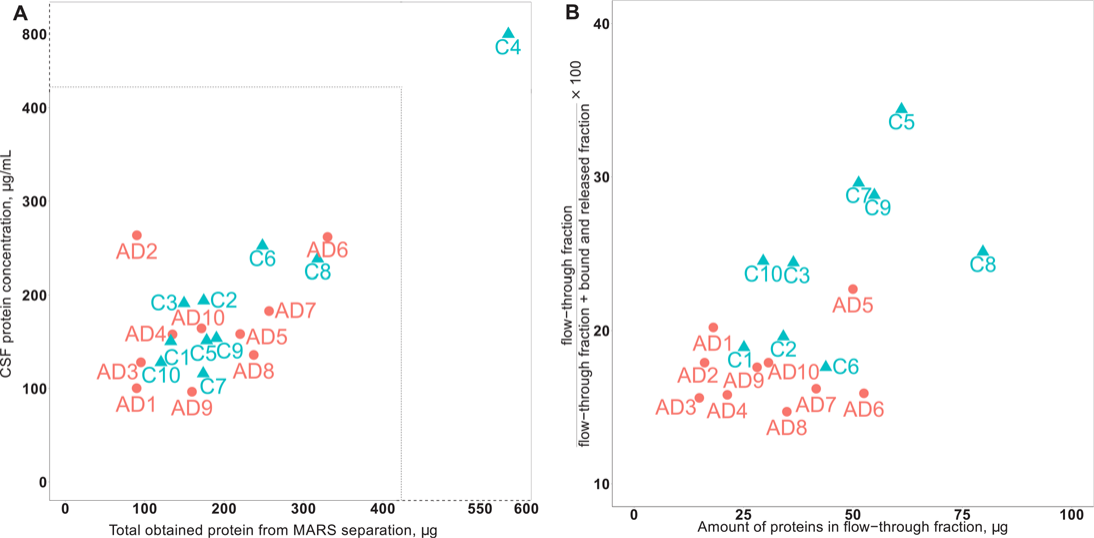
Protein concentration in AD and control samples before and after hu-7 depletion. (A) Total amount of protein in CSF in AD and control samples (a control sample showed aberrantly high protein amount and was omitted in further protein amount calculation). (B) Percentage of proteins left after hu-7 depletion showed a statistical significant difference (p = 0.003) between the two groups. AD: Alzheimer’s disease; C: healthy control.

The protein amount in the depleted CSF fraction (the flow-through fraction) was 46.2±17 μg and 30.8±14 μg for the control (n = 9) and AD patient (n = 10) group, respectively, showing a statistically significant difference (p-value = 0.046). Calculation of the fraction between unbound and bound proteins showed that 24.8±5.5% and 17.5±2.4% was obtained in the depleted CSF fraction for the control and AD patient groups, respectively. The difference between the groups was statistically significant (p-value = 0.003). No significant difference was found for the amount of proteins captured and released by the column (Fig 1).

### Software comparisons

#### Peptide identification

**Fig 2:**
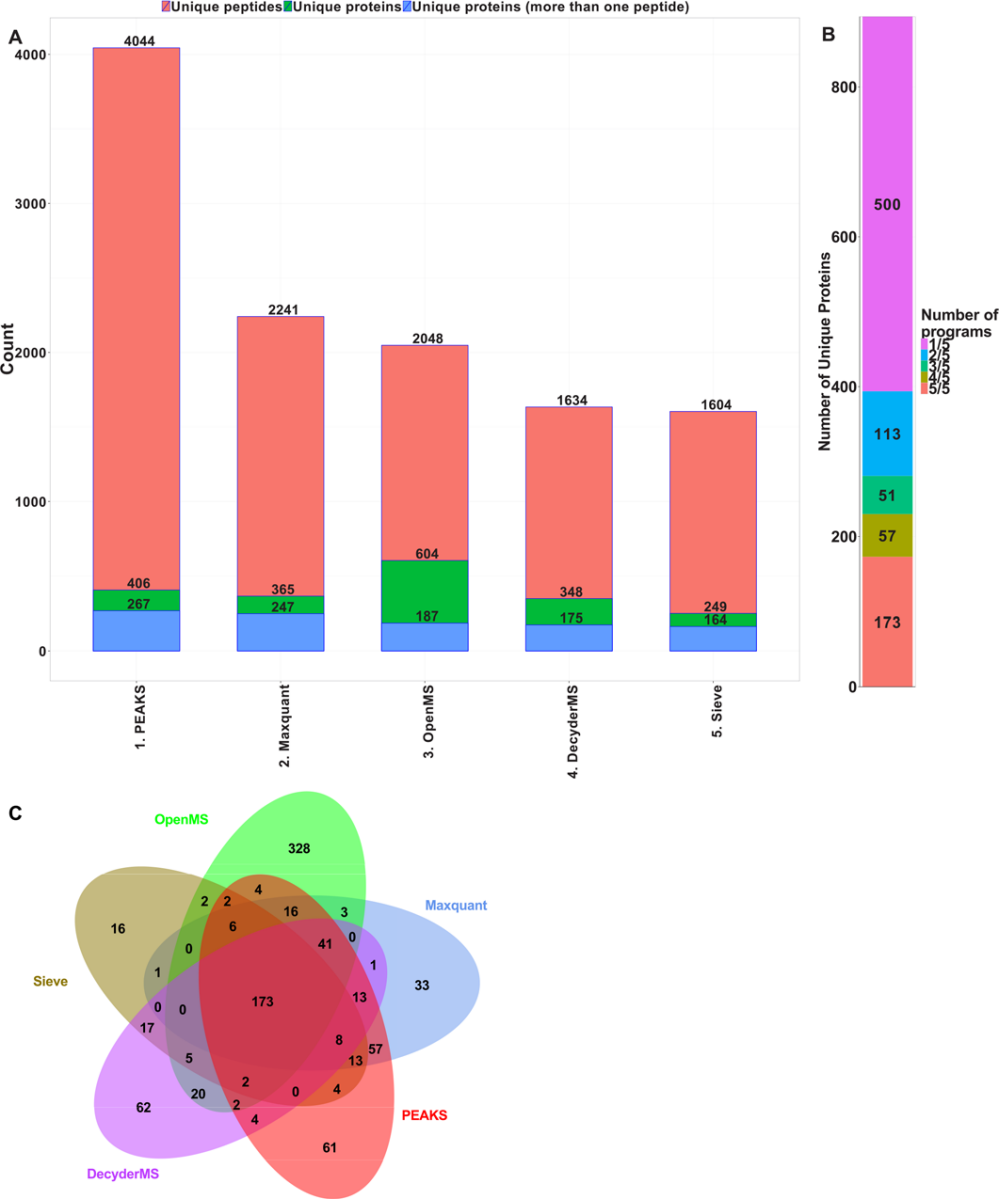
Five data processing programs identification comparison. (A) Comparison of identification performance between five mass spectrometry data processing tools based on number of identified peptides, proteins, and proteins identified with more than one peptide. (B) The number of identified and mapped proteins across the different programs. (C) Overlap of protein identification between different programs.

The highest number of unique peptides was identified and mapped using PEAKS (Fig 2A). The number of identified and mapped peptides in PEAKS was almost twice as many compared to the other programs. Similar identification performance was observed using Maxquant and OpenMS. The lowest number of identified peptides was found using Sieve and DecyderMS. The highest number of proteins was identified using OpenMS, but PEAKS identified the highest number of proteins characterized with more than one peptide. The lowest number of proteins was mapped and identified using Sieve and DecyderMS. Only 173 proteins out of 894 proteins was identified and mapped across all five programs whereof the majority of the proteins were identified and mapped by only one program (Fig 2B and 2C).

#### Reproducibility

The highest correlation between the technical replicates was found using Sieve (median R^2^ = 0.981). OpenMS (median R^2^ = 0.971), PEAKS (median R^2^ = 0.971), and DecyderMS (median R^2^ = 0.971) performed the second best. Maxquant (median R^2^ = 0.911) showed substantially lower correlations between the technical replicates compared to other programs. The variance ratio comparison also showed that Sieve (median ratio = 1.001) had the lowest ratio of variation between the technical replicates, followed by OpenMS (median ratio = 1.003), DecyderMS (median ratio = 1.004), PEAKS (median ratio = 1.033), and Maxquant (median ratio = 0.976) (S1A and S1B Fig).

**Fig 3:**
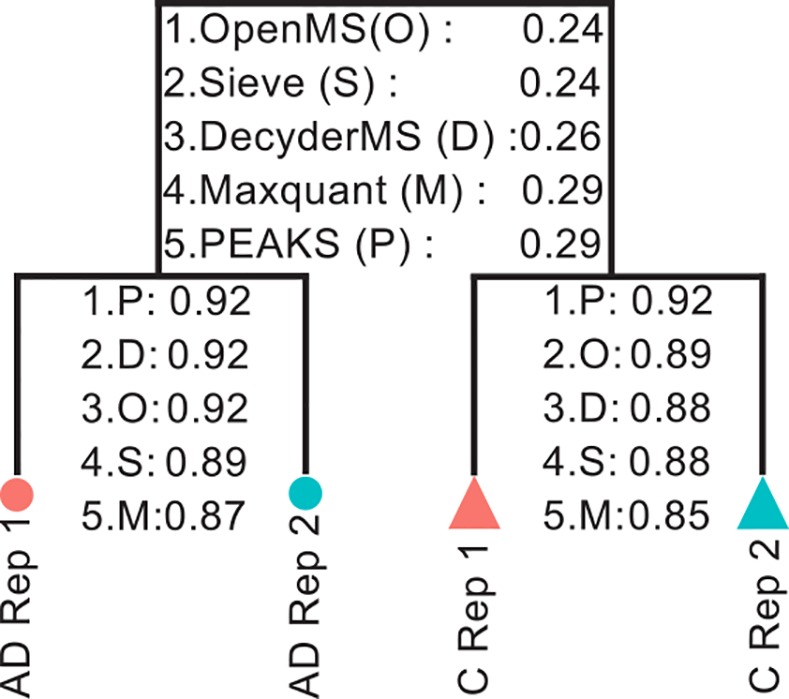
Technical replicates reproducibility comparison. Five programs were ranked based on the distance between technical replicates as well as between AD and control samples. The p-values were calculated based on Mahalanobis distance computed on the first two components of PCA (for raw data in each program). Lower p-value reflects less similarity between the groups (AD and controls as well as between technical replicates). The highest distance between AD and controls was found using OpenMS and the lowest distance between technical replicates was found using PEAKS. Rep: replicate; AD: Alzheimer’s disease; C: healthy controls.

In general, we observed high reproducibility between replicates as indicated by clear clustering of technical replicates using PCA (S2 Fig). However, in terms of Mahalanobis distance between the first two component of PCA, the highest reproducibility (higher p-value) was found using PEAKS and the lowest reproducibility was found using Maxquant (Fig 3). Furthermore, assuming a difference between AD and controls, OpenMS and Sieve showed slightly better separation between AD and controls compared to the other programs.

#### Disease-associated proteins

Using the combined results of all the five programs and after manual curation, we found 162 statistically significantly altered proteins (p-value<0.05) between Alzheimer’s disease and non-demented controls (S3 Table) where 31 proteins was identified and found to be statistically significantly differentially altered by all the five programs.

#### Effect of normalization on quantification

**Fig 4:**
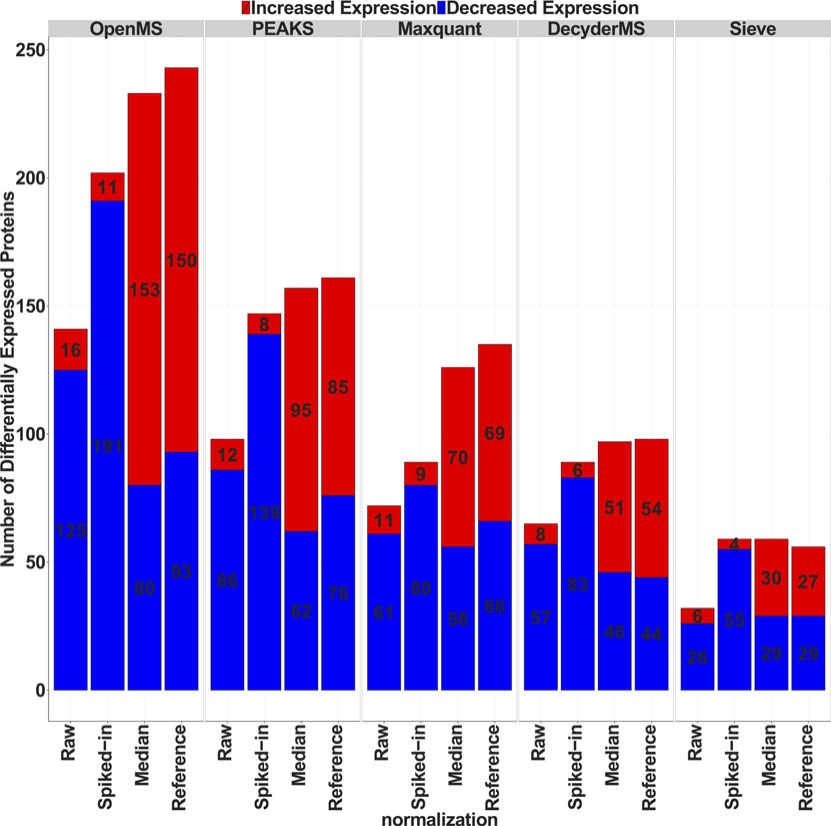
Comparison of proteins with significantly changing levels between AD and healthy controls. Comparison of the number of significantly altered proteins identified by different normalization methods and five mass spectrometry data processing tools.

In order to examine the effect of normalization methods on the number of statistically significantly altered proteins between AD and healthy controls, we compared the number of proteins with positive and negative fold changes in each program after applying each normalization method. Using the median or reference normalization, nearly the same proportion of the proteins with increased and decreased level was statistically significantly altered across all the programs. However, when the normalization was performed locally (spiked in normalization), the number of statistically significantly altered proteins with increased level was decreased and the number of proteins with decreased level was increased in all the programs (Fig 4). The overlap of significantly altered proteins between the programs was low using non normalized data (S3 Fig). Higher overlap was found using spiked-in and median normalization (S3B and S3C Fig) compared to reference normalization (S3D Fig).

#### Verification using antibody-based profiling

At the time of analysis and to the best of our knowledge, 70 out of the 162 proteins have previously not been reported to be statistically significantly altered in levels between AD and healthy controls. Based on antibody availability in the Human Protein Atlas, 52 proteins (represented by in total 112 antibodies) were selected for analysis using the suspension bead array technology. After initial data quality control, one of the AD samples was excluded from further analysis.

**Fig 5:**
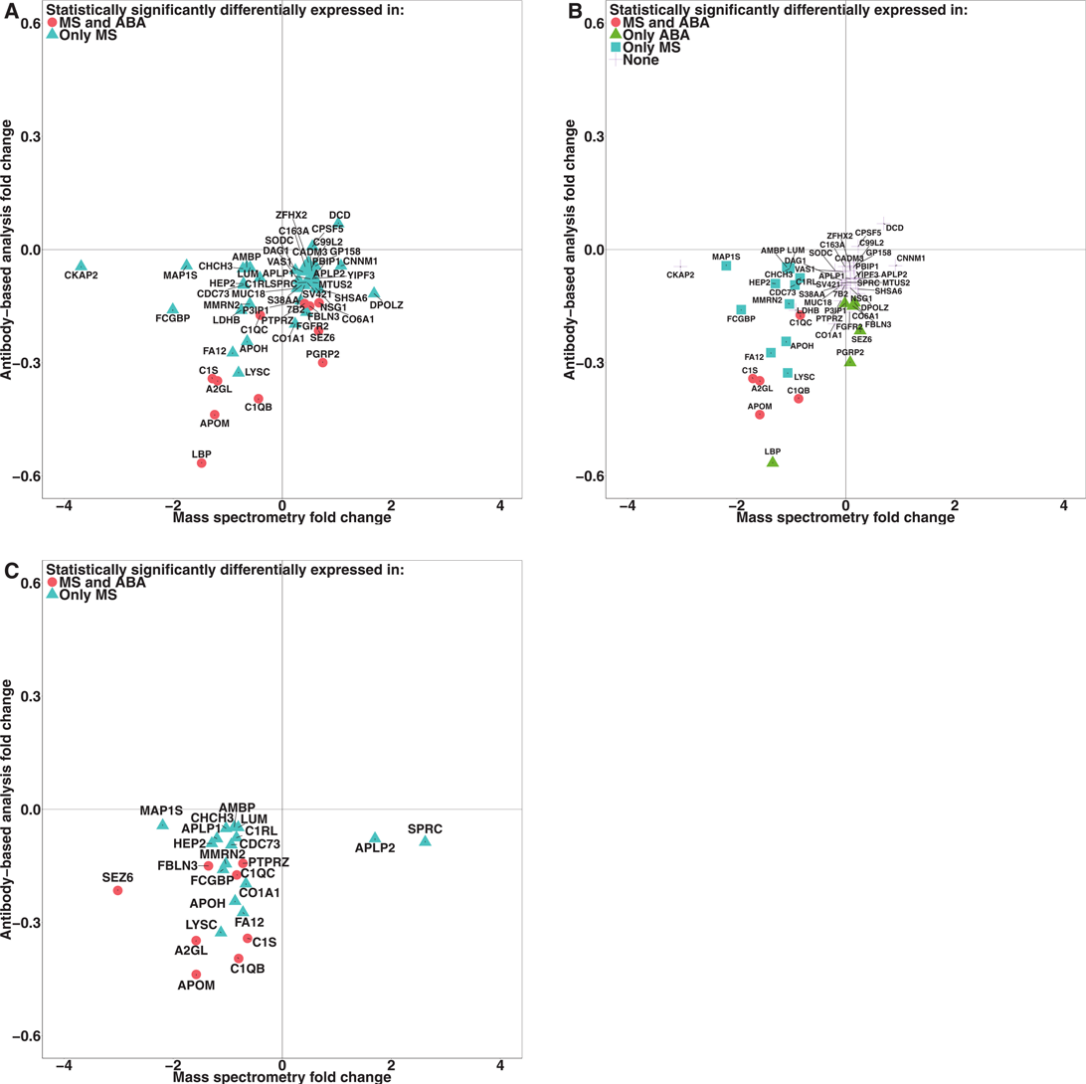
Comparison of protein fold changes derived from the antibody-based analysis and mass spectrometry. (A) Scatter plot of protein fold changes between mass spectrometry (reference normalization) and antibody-based analysis. The protein p-values and fold changes were calucluated using statistically significantly differentially altered peptides. (B) Scatter plot of protein fold changes between mass spectrometry (spiked-in normalization) and antibody-based profiling. After normalization with spiked-in method, the protein p-values and fold changes were computed using the same peptides as used in the panel A (C) Scatter plot of protein fold changes between mass spectrometry (spiked-in normalization) and a where the protein p-values and fold changes were computed using statistically significantly differentially altered peptides (proteins are shown based on Uniprot ID). MS: Mass spectrometry; ABA: antibody-based analysis.

**Fig 6:**
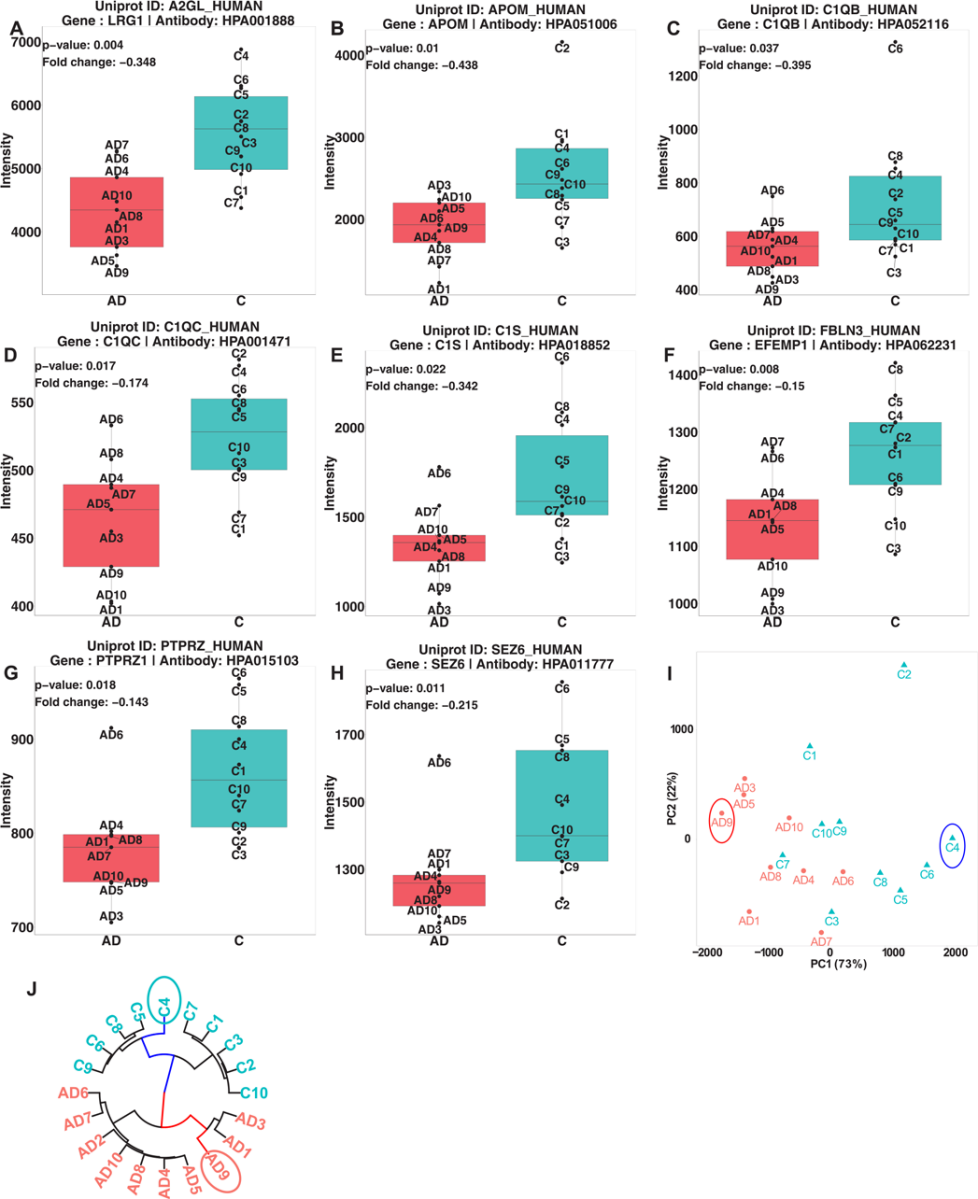
Quantitative analysis of proteins from antibody-based technique. (A)-(H) Relative intensities of the eight disease-associated proteins. (I) Principal component analysis of the eight proteins (J) clustering of the sample in MS analysis (only openMS is shown) where C4 and AD9 samples are deviating from the other samples. LRG = Leucine-rich alpha-2-glycoprotein; APOM = Apolipoprotein M; C1QB = Complement C1q subcomponent subunit B; C1QC = Complement C1q subcomponent subunit C; C1S = Complement C1s subcomponent; FBLN3 = EGF-containing fibulin-like extracellular matrix protein 1; PTPRZ = Receptor-type tyrosine-protein phosphatase zeta; SEZ6 = Seizure protein 6 homolog.

The antibody-based analysis revealed 11 of the 52 proteins (S4 Table) as statistically significantly different (p-value<0.05) between AD and healthy controls in two repeated experiments with a median technical variation of 5%. For the majority of the proteins we found decreased level in AD samples compared to healthy controls. Fold change comparison of mass spectrometry (reference normalized data) and antibody-based profiling measurements revealed inconsistencies between the two techniques in the direction of the fold changes of 30 out of 52 targeted proteins (Fig 5A). However, when the data was normalized using spiked-in normalization method and the same peptides (as used for reference normalization) were used to compute the fold changes and p-values, we found that the fold changes for a majority of the proteins with inconsistencies were reduced to 20 proteins (Fig 5B). Furthermore, with the spiked-in normalization the number of statistically significant proteins was decreased from 52 to 17 proteins. Overall, with this method there were consistent fold changes between mass spectrometry and antibody-based profiling for 22 out of 24 proteins (Fig 5C). Taking both MS and antibody-based profiling into account, eight proteins were statistically significant (Table 2). Among the eight proteins, four proteins were found to be significantly differentially altered by at least two programs and the remaining proteins were only found by one program (Table 2). These proteins included leucine-rich α2 glycoprotein (LRG), apolipoprotein M (ApoM), complement C1q subcomponent (subunit B, C) (C1QB and C1QC), сomplement C1S (C1S), EGF-containing fibulin-like extracellular matrix protein 1 (fibulin-3, FIBL3), receptor-type tyrosine-protein phosphatase zeta (PTPRZ) and seizure protein 6 homolog (SEZ6), all displaying lower levels in AD compared to controls (Fig 6A–6H). Additionally, when the level of eight proteins in antibody-based analysis was transformed using PCA, we observed that the control sample with the abnormal concentration (labeled as C4) was clearly deviating from the other samples as well as the AD sample (AD9) with the longer duration of the disease (Fig 6I). Moreover, nearly the same pattern of deviation was found in the clustering of significantly differentially abundant peptides/peaks in the MS data (Fig 6J).

**Table 2:** Proteins selected from spiked-in normalized data that were verified in the antibody-based analysis as novel disease-associated markers for Alzheimer’s disease.

	Antibody based analysis			Mass spectrometry					
Uniprot ID	Antibody used for statistical analysis	p-value	Fold change	Median p-value	Fisher’s p-value	Median fold change	Highest correlated statistically significantly differentially expressed peptide to the significantly differentially expressed antibody	Correlation of the peptide to the statistically significantly differentially expressed antibody	Statistically significantly differentially altered in program
A2GL_HUMAN	HPA001888	0.004	-0.348	0.0001	<0.0001	-1.579	LQELHLSSNGLESLSPEFLRPVPQLR	0.972	DecyderMS—Maxquant—**OpenMS**—PEAKS—Sieve
APOM_HUMAN	HPA051006	0.010	-0.438	0.021	0.021	-1.577	SLTSC(Carbamidomethyl)LDSK	0.733	**OpenMS**
C1QB_HUMAN	HPA052116	0.037	-0.395	0.027	<0.0001	-0.800	LEQGENVFLQATDK	0.912	DecyderMS—Maxquant—**OpenMS**—PEAKS—Sieve
C1QC_HUMAN	HPA001471	0.017	-0.174	0.030	0.001	-0.834	VPGLYYFVYHASHTAN(Deamidated)LCVLLYR	0.810	DecyderMS—Maxquant—**OpenMS**—PEAKS—Sieve
C1S_HUMAN	HPA018852	0.022	-0.342	0.027	<0.0001	-0.637	VEDPESTLFGSVIR	0.938	DecyderMS—**OpenMS**—PEAKS—Sieve
FBLN3_HUMAN	HPA062231	0.008	-0.150	0.044	0.044	-1.353	EHIVDLEMLTVSSIGTFR	0.857	**Maxquant**
PTPRZ_HUMAN	HPA015103	0.018	-0.143	0.049	0.049	-0.721	AIIDGVESVSR	0.639	**DecyderMS**
SEZ6_HUMAN	HPA011777	0.011	-0.215	0.004	0.004	-3.015	RPAYGDVTVTSLHPGGSAR	0.890	**DecyderMS**

The presented peptide sequences represent peptides with the highest correlation to the corresponding antibody. The mass spectrometry statistical information was found using the program shown in bold text in the corresponding column.

#### Correlation to antibody-based profiling

**Fig 7:**
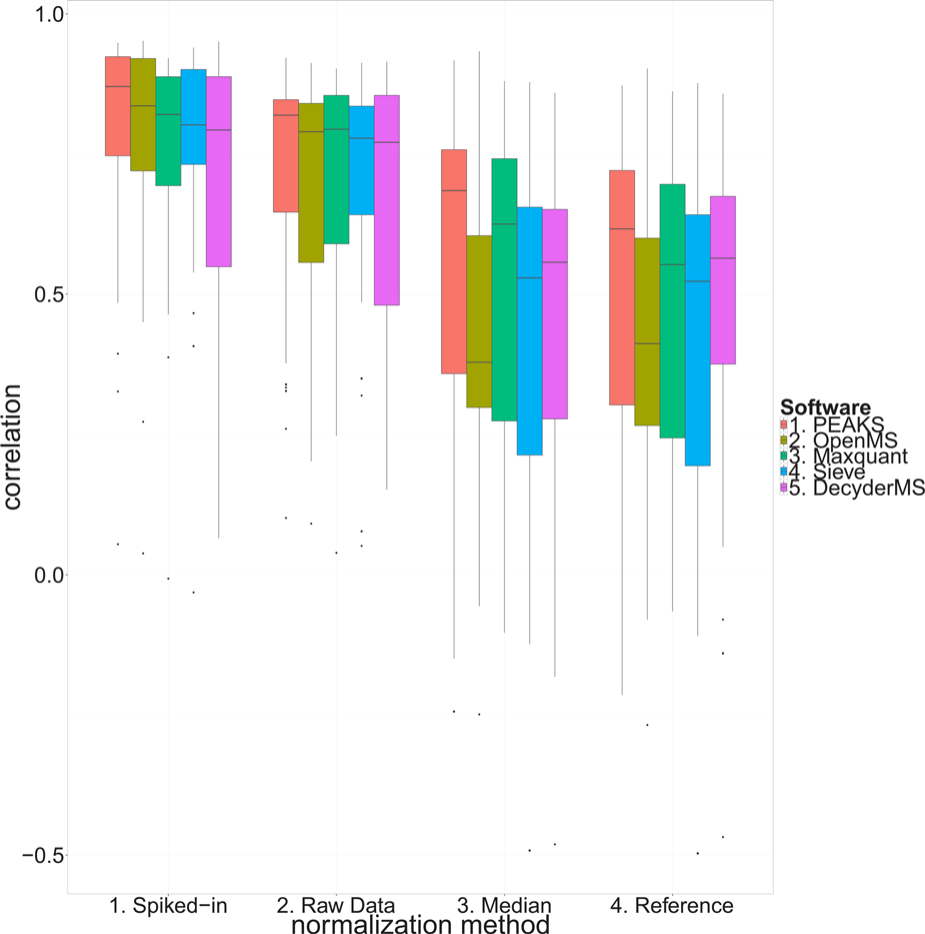
Distribution of all the highest correlated peptide-antibody pairs between mass spectrometry and antibody based analysis. The results obtained for the five programs and three normalization methods used were correlated to the antibody-based analysis.

Out of 52 targeted proteins, 27 proteins were identified and quantified by all the programs (S5 Table, bold and underlined entities represent the overlapping proteins across the programs). Comparing correlation distribution of the overlapping proteins from the three normalization methods revealed that performing local normalization (spiked-in) resulted in a considerable improvement of correlations between the mass spectrometry and antibody-based profiling measurements compared to raw data and global normalization methods (median correlation for each normalization: spiked-in: 0.826, raw data: 0.794, reference: 0.542, median: 0.553) irrespective of the software used for quantification (Fig 7; an example for correlation improvement is illustrated in S4 Fig). Furthermore, quantification using raw data led to higher correlations compared to the global normalization methods (reference and median normalization). The reference and median normalization methods resulted in similar correlations between mass spectrometry and antibody-based profiling measurements. Moreover, we observed similar correlation patterns in all the programs (median correlation using spiked-in normalization: PEAKS: 0.870, OpenMS: 0.836, Maxquant: 0.820, Sieve: 0.802, DecyderMS: 0.793). The same overall pattern of correlation was found when the programs were compared using all the proteins (S5 Fig).

## Discussion

### Lower fraction of low-abundance proteins in Alzheimer’s disease

After depleting of the seven most abundant proteins from the CSF samples, the protein amount and the fraction of proteins in the unbound depleted fraction in the subject with AD was statistically significantly lower compared to the healthy elderly controls. The lower protein amount might reflect loss of several proteins due to the depletion procedures [46] but since the samples were randomized before depletion the possibility of protein loss only in the AD group is low, but it can not be ruled out. The difference in protein amount might also represent the age difference between the study groups [10], where the elderly healthy controls are on average nine years older compared to AD. However, previous studies are suggesting novel CSF AD biomarkers with lower levels in AD CSF compared to controls [47–51] that might reflect more general differences in protein amount between AD and non-AD.

### Effects of initial data analysis on peptide identification and reproducibility

There are several programs developed for initial mass spectrometry data processing and protein identification. However, due to the different algorithms implemented in these programs, they might produce different and even contradictory results [23]. Multiple studies have evaluated and compared different data processing and identification algorithms used in these programs [52–57]. We found that the PEAKS program identified and mapped considerably higher number of peptides compared to the other programs. PEAKS uses a combination of three identification search engines through the PEAKS database (a de novo sequencing database) [35]. It has previously been reported that combining multiple search engines substantially increases the number of identified peptides and proteins [58], a finding in line with our observation that both PEAKS and OpenMS identified more peptides than Sieve and DecyderMS (which both use the Mascot search engine). Despite identifying substantially higher number of peptides using PEAKS, the number of identified proteins was similar to the other programs. We found that PEAKS search engine is capable of characterizing each unique protein by multiple peptides. In addition, we found that Maxquant identified higher number peptides compared to Sieve and DecyderMS which is in line with other studies by Cox, J., et al [32] and Colaert, N., et al using SILAC [59]. However, we found that many of the peptides identified only in Maxquant (not in OpenMS) were in fact non-proteotypic peptides (the peptides that were assigned to more than one protein). After removing these peptides, the peptide identification performance was similar between the two programs. Finally, observed that the majority of the proteins were identified by one program. This can be explained by multiple factors such as different pre-processing methods, scoring functions as well as failure to build a mass feature for the peptides (unmapped peptides). This implies that to gain appropriate peptide/protein coverage, multiple programs can be employed not only for the identification but also for the quantification. As for identification there are several methods to score combined identification from multiple search engines [38]. However, to the best of our knowledge there are no approaches to score or combine quantification results from several programs.

In label free shotgun proteomics, it is common to make technical replicates for each sample to increase the reliability of the downstream analysis. In general, all the programs produced acceptable correlation and low variation between the technical replicates also indicating high reproducibility of the MS analysis. Sieve produced the highest reproducibility and in agreement with previous studies, we also found that OpenMS produced higher reproducibility across technical replicates compared to Maxquant [60] as well as DecyderMS and PEAKS. Considering the variation ratio, PEAKS program was deviating from the other programs whereas it resulted in similar correlation pattern to the other programs, achieving higher correlation than DecyderMS and Maxquant. We found that these high correlations were caused by a number of peptides with large intensities and high correlation between the technical replicates that masked the low correlation of peptides with small abundance. Therefore, despite having high correlation between the technical replicates, variance ratio comparison resulted in large difference in variance in some of the technical replicate pairs in PEAKS program.

### Data normalization affects biological conclusions

Normalization is performed to reduce the technical variation and remove bias caused by differences in protein concentration and other technical aspects of the MS analysis [40, 61]. Using relative protein levels generated by antibody-based profiling, we compared the mass spectrometry results using three different normalization methods (median, reference, and spiked-in normalization) based on data from five different data processing programs. We found that global normalization strategies resulted in low correlation to the affinity data. Most of the global normalization methods are based on the assumption that the distribution of proteins with increased and decreased abundance between groups of interest is nearly symmetrical [26]. The violation of this assumption in the investigated CSF samples was not clear until removal of the seven most abundant proteins prior to MS analysis, which is contributing with more than 75% on the total protein mass. By performing local normalization based on a spiked-in protein, thus only correcting for experimental bias, the correlations to the antibody-based profiling results were substantially increased. It is important to note that the antibody-based profiling was performed on the crude and not depleted CSF. Spiked-in normalization resulted in a considerable reduction of fold changes comparing AD to non-AD, a more consistent result to that of antibody-based technique and this was found irrespective of the program used for the MS quantification. In addition, spiked-in normalization resulted in equal or higher overlap of result between the programs (despite finding lower number of altered proteins). This suggests that global normalization should be used with care when analyzing CSF in case of systematic differences between groups of interest can not be ruled out. We suggest adding one or several recombinant proteins from a different species than the investigated, which can be used for correcting for experimental bias and to investigate if the assumption for using global normalization is valid.

### Disease associated proteins

Eight proteins were found to display concordant results using the two technologies, all displaying lower levels in the AD patients compared to the controls. Several of the reported proteins, including ApoM, LRG, FBLN3 and PTPRZ, have functions related to cell adhesion, migration, and morphology [62–71] and have been reported as important in the development of various cancer types [72–79] as well as diabetes [80, 81]. Also related to immune system, (C1QB and C1QC), complement C1S, and SEZ6 have been reported to be involved in synapse development [82–84]. However, the relation of these proteins to the neurological diseases is not understood. We are conducting a study with a large cohort to further verify the presence of these proteins in CSF.

## Supporting Information

S1 DataFile Antibody based technique quantification data. (XLSX)

S2 DataFile DecyderMS median normalized data quantification data. (XLSX)

S3 DataFile DecyderMS raw data quantification data. (XLSX)

S4 DataFile DecyderMS reference normalized data quantification data. (XLSX)

S5 DataFile DecyderMS spiked in normalized data quantification data. (XLSX)

S6 DataFile Maxquant median normalized data quantification data. (XLSX)

S7 DataFile Maxquant raw data quantification data. (XLSX)

S8 DataFile Maxquant reference normalized data quantification data. (XLSX)

S9 DataFile Maxquant spiked in normalized data quantification data. (XLSX)

S10 DataFile OpenMS median normalized data quantification data. (XLSX)

S11 DataFile OpenMS raw data quantification data. (XLSX)

S12 DataFile OpenMS reference normalized data quantification data. (XLSX)

S13 DataFile OpenMS spiked in normalized data quantification data. (XLSX)

S14 DataFile PEAKS median normalized data quantification data. (XLSX)

S15 DataFile PEAKS raw data quantification data. (XLSX)

S16 DataFile PEAKS reference normalized data quantification data. (XLSX)

S17 DataFile PEAKS spiked in normalized data quantification data. (XLSX)

S18 DataFile Sieve median normalized data quantification data. (XLSX)

S19 DataFile Sieve raw data quantification data. (XLSX)

S20 DataFile Sieve reference normalized data quantification data. (XLSX)

S21 DataFile Sieve spiked in normalized data quantification data. (XLSX)

S22 DataFile Identified and mapped (to a feature) proteins and peptides using DecyderMS software. Charge state and mass/charge are not available for DecyderMS since the program combines the charge states.
(XLSX)

S23 DataFile Identified and mapped (to a feature) proteins and peptides using Maxquant software. (XLSX)

S24 DataFile Identified and mapped (to a feature) proteins and peptides using OpenMS software. (XLSX)

S25 DataFile Identified and mapped (to a feature) proteins and peptides using PEAKS software. (XLSX)

S26 DataFile Identified and mapped (to a feature) proteins and peptides using Sieve software. (XLSX)

S1 Fig Reproducibility comparison between five data processing programs. (A) Distribution of coefficient of determination between the technical replicates in five mass spectrometry data processing programs. The higher the correlation the closer the replicates quantification. (B) Distribution of variation ratios between the technical replicates in each tool. The closer the values to 1 the lower the variation between the technical replicates.
(PDF)

S2 Fig Reproducibility of mass spectrometry experiment. PCA of peptide intensities showing how study groups (AD: Alzheimer’s disease; C: healthy control) and the technical replicates (the number after underline) are clustered. (A) DecyderMS. (B) Maxquant. (C) OpenMS. (D) PEAKS. (E) Sieve.
(PDF)

S3 Fig Overlap of significantly altered proteins between different programs using raw data and three normalization methods. (A) Raw data. (B) Spiked-in normalization. (C) Median normalization. (D) Reference normalization.
(PDF)

S4 Fig An example of correlation improvement using three normalization method and raw data. Scatter plot of the highest correlated peptide between mass spectrometry and luminex (Protein P3IP1). Protein names are shown as Uniprot ID. (A) Reference normalization. (B) Median normalization. (C) Raw data. (D) Spiked-in normalization. Abb. ABA: antibody-based analysis.
(PDF)

S5 Fig Distribution of all the highest correlated peptide-antibody pairs between mass spectrometry and antibody-based analysis. The results obtained for the five programs and three normalization methods used were correlated to the antibody-based analysis.
(PDF)

S1 Table Depletion setup for CSF samples. (XLSX)

S2 Table LC-MS/MS run order CSF samples. (XLSX)

S3 Table Differentially altered proteins. (XLSX)

S4 Table The proteins with statistically significantly altered levels in the antibody-based profiling. (XLSX)

S5 Table Correlation of the highest correlated peptide-antibody pair between Mass spectrometry and antibody-based technique. (XLSX)
