# Supplementary figures and images for: Analysis of the Cerebrospinal Fluid Proteome in Alzheimer's Disease
Source: PLoS One. 2016 Mar 7;11(3):e0150672. doi: 10.1371/journal.pone.0150672 (PMC4780771; doi:10.1371/journal.pone.0150672)

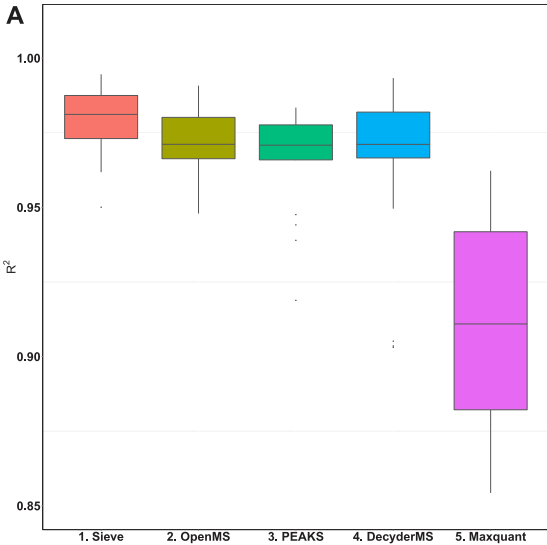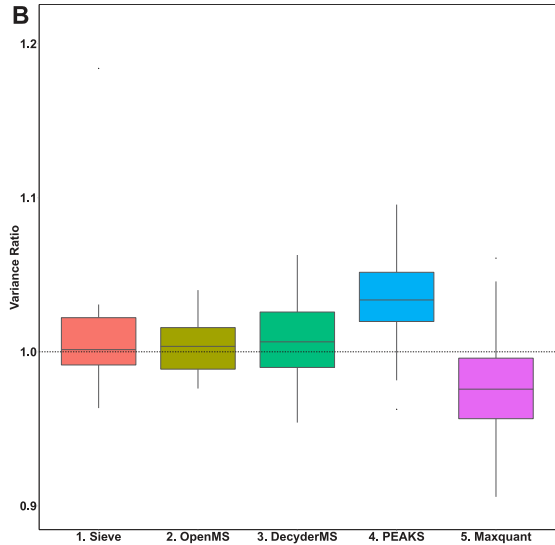

Supplement: S1 Fig — (A) Distribution of coefficient of determination between the technical replicates in five mass spectrometry data processing programs. The higher the correlation the closer the replicates quantification. (B) Distribution of variation ratios between the technical replicates in each tool. The closer the values to 1 the lower the variation between the technical replicates. (PDF) [file pone.0150672.s027.pdf]

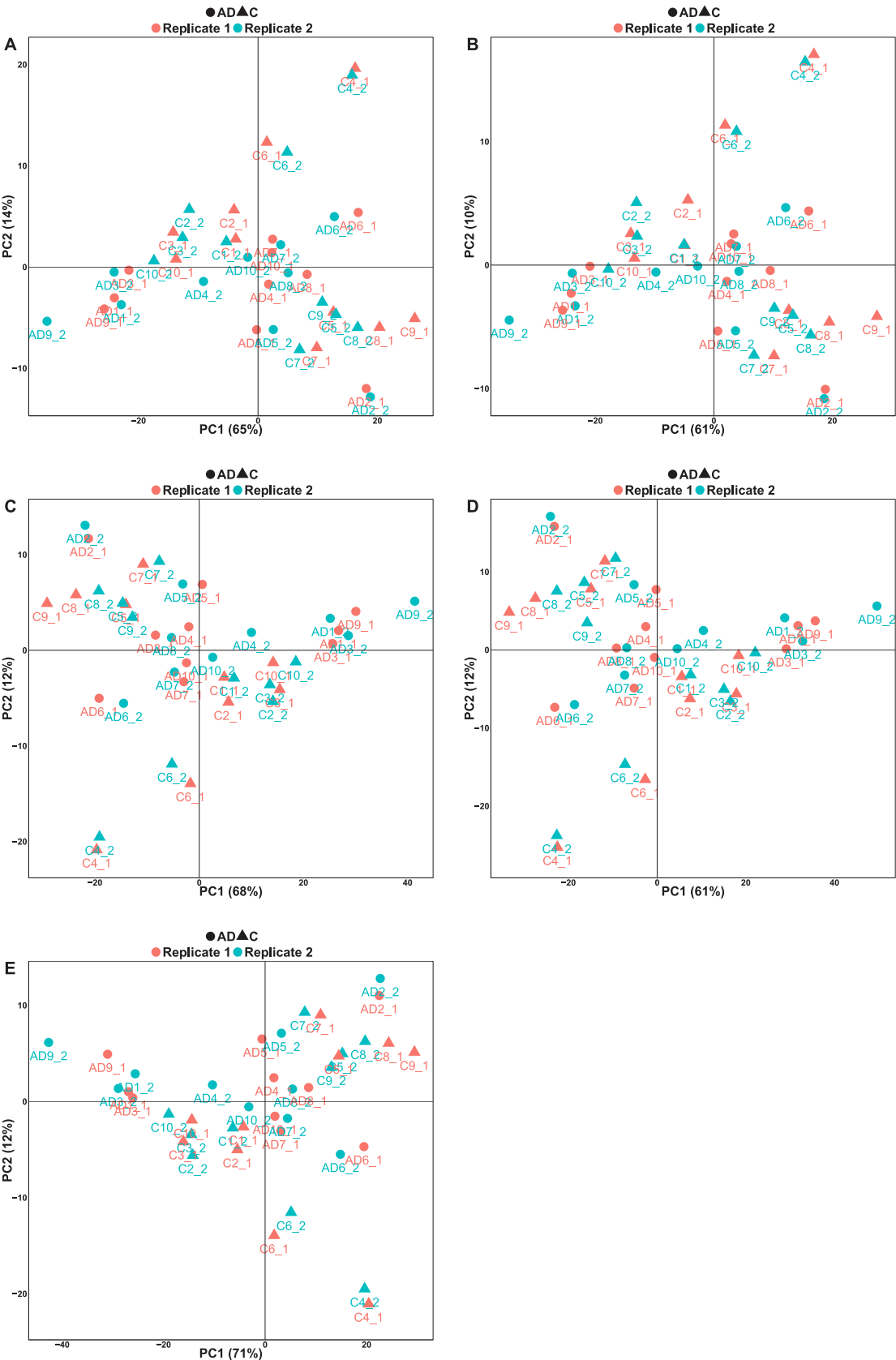

Supplement: S2 Fig — PCA of peptide intensities showing how study groups (AD: Alzheimer’s disease; C: healthy control) and the technical replicates (the number after underline) are clustered. (A) DecyderMS. (B) Maxquant. (C) OpenMS. (D) PEAKS. (E) Sieve. (PDF) [file pone.0150672.s028.pdf]

## Sieve

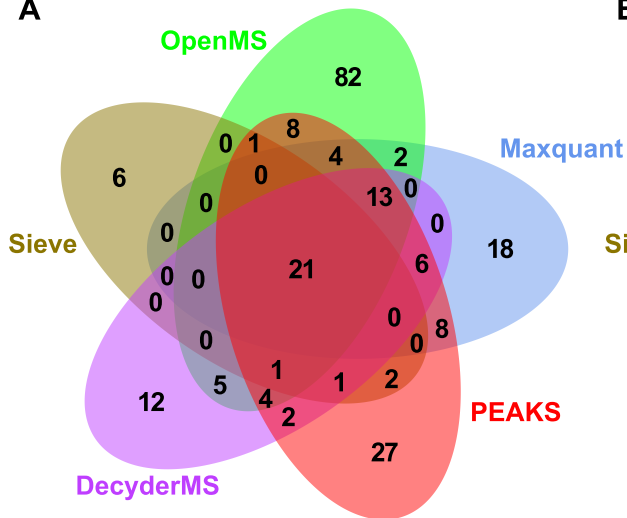

## Sieve

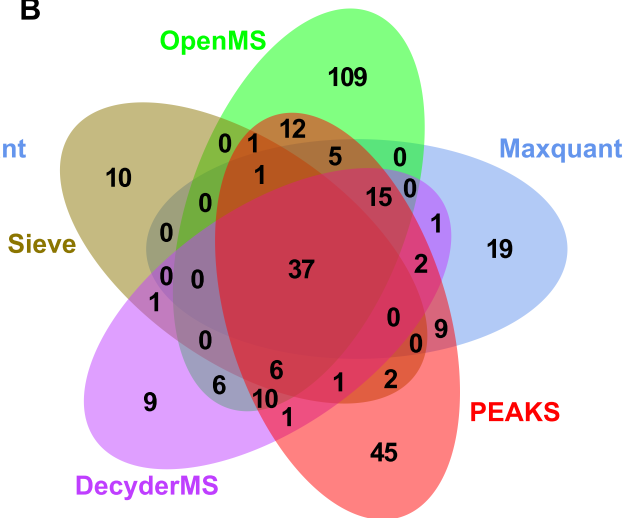

## Sieve

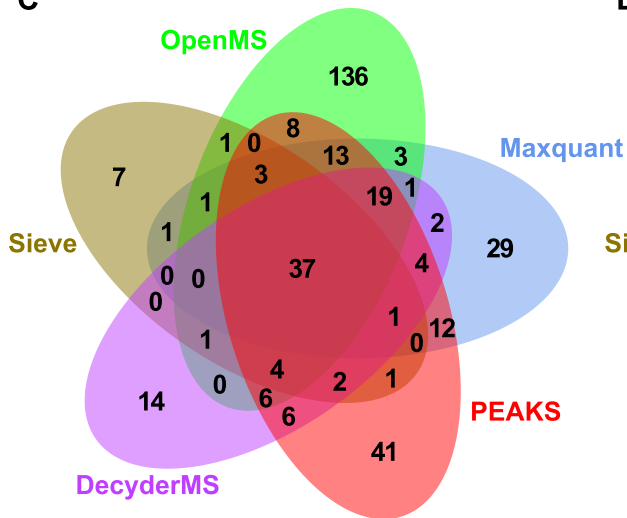

## Sieve

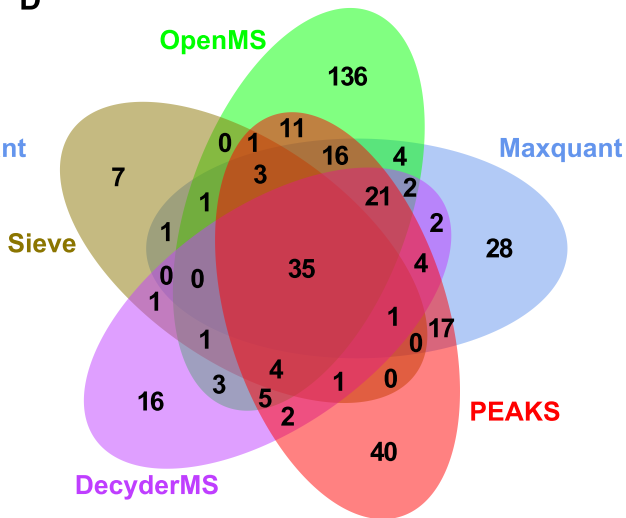

Supplement: S3 Fig — (A) Raw data. (B) Spiked-in normalization. (C) Median normalization. (D) Reference normalization. (PDF) [file pone.0150672.s029.pdf]

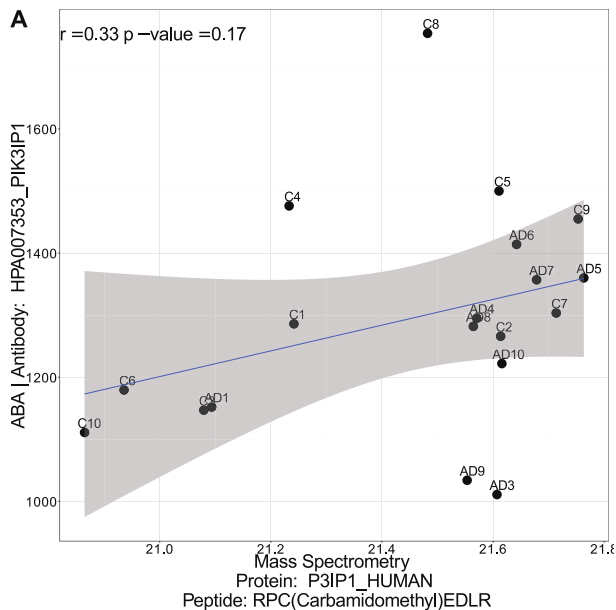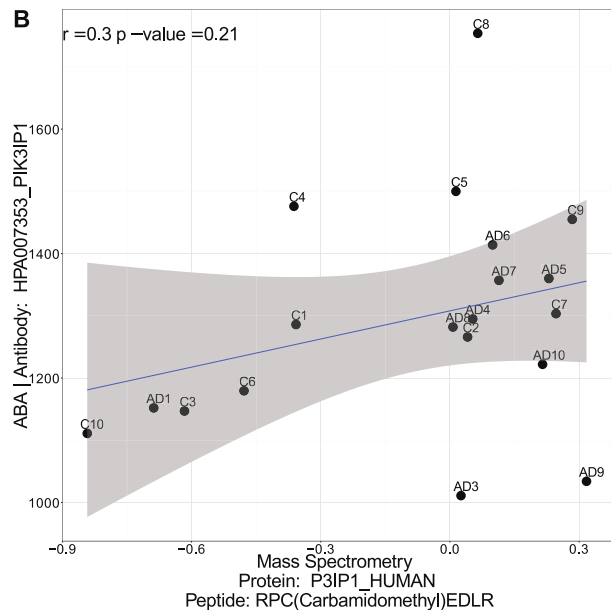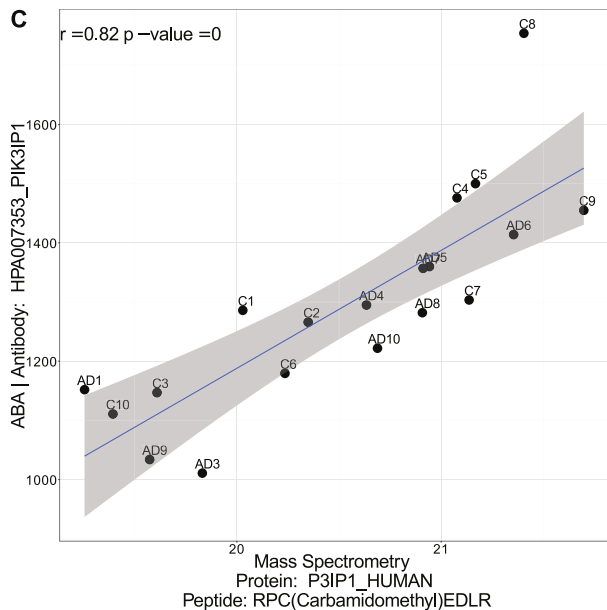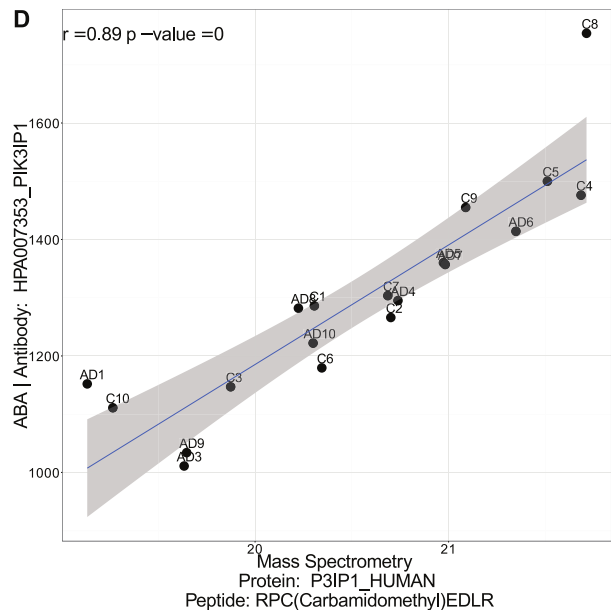

Supplement: S4 Fig — Scatter plot of the highest correlated peptide between mass spectrometry and luminex (Protein P3IP1). Protein names are shown as Uniprot ID. (A) Reference normalization. (B) Median normalization. (C) Raw data. (D) Spiked-in normalization. Abb. ABA: antibody-based analysis. (PDF) [file pone.0150672.s030.pdf]

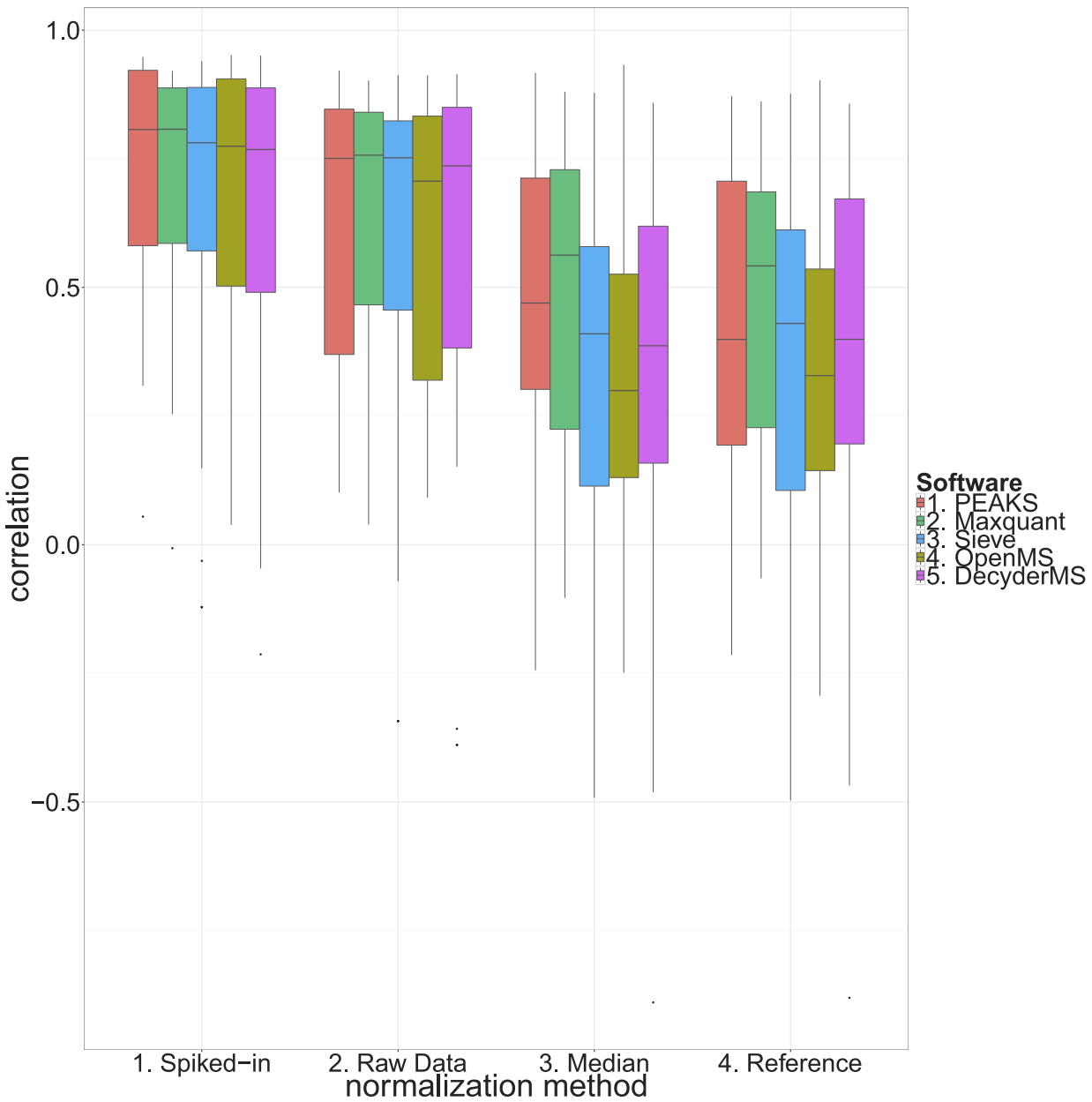

Supplement: S5 Fig — The results obtained for the five programs and three normalization methods used were correlated to the antibody-based analysis. (PDF) [file pone.0150672.s031.pdf]
